# The First Christmas after the Armistice

**Published:** 1918-12-28

**Authors:** 


### THE WORST WAR OF THE WORLD.

### VII. THE FIRST CHRISTMAS AFTER THE ARMISTICE.

Christmas Day, 1918, the first Christmas after the cessation of the actual fighting in the Great War, so far as the Allies are concerned at the moment, will be remembered as unique when compared with all preceding Christmases up to and including that of 1914. It will be known as Victory Christmas, for the celebration of which very special and universal preparations have been made throughout Great Britain, in which the Navy and Army authorities, and those of the Red Cross, the Hospitals and all associated with the treatment and care of the sick have combined, so that to the utmost extent possible family reunion shall prevail in every household throughout the land. Not the least welcome of these manifestations of unity, thankfulness, and rejoicing has been the return of a portion of the Fleet and distinguished Admirals, though the great demonstration by the Fleets of the Allies and the welcome of the people of the Empire to the British Fleet when the day arrives and Peace has been declared, will move the whole world to recognise and render for ever memorable, the first after-war review of our Fleets and sea-craft.

The Army, now in its fifth year of continuous conflict, struggle, and heavy fighting, to whose invariable and splendid courage, staying powers, and grit we owe so much of our liberties and the victories achieved in God's mercy, has been fortunate enough to anticipate Christmas 1918 by the return of Field-Marshal Sir Douglas Haig and his great Generals, Sir William Birdwood, Sir Henry Rawlinson, Sir Herbert Plumer, Sir H. S. Horne, and Sir Julian Byng, who were received and entertained by the King on the 19th instant at Buckingham Palace, and whose portraits we hope to give in our next issue. They have had the good fortune to return home and celebrate their Christmas with their families, so that they may unite in harmony with every British family in the kingdom, and join with them in grateful thanksgiving for the wonderful mastery which has been granted to our Fleets, our Armies, and Airmen, and those of our Allies, in this, the greatest war of the world.

Some of the papers have been full of impatient declamation because the demobilisation of our fighting forces, and the return of our warriors to their homes and ordinary occupations, have not so far proceeded at the pace which these impatient and, in the large sense of the word, discredited complainants in the press and elsewhere have been pleased, without adequate reason or ordinary justice or prudence, to consider desirable. Demobilisation has been prepared for and will proceed with all possible expedition, having regard to the enormous responsibilities which attach to it, and the impossibility at the present time of obtaining the clear issues, which can alone become practicable, when there are created to demonstration representative authorities accepted by the nations which collectively constitute our opponents in the war. There is not a man among the Allies, from the greatest to the humblest, who would not gratefully welcome the day when everything is sufficiently forward and clear to enable the Peace Conference to assemble, and the serious business of Peace to arrive at a basis, in every field of war, upon which a genuinely permanent and enduring peace can be carried through to a satisfactory conclusion.

This moment has not yet arrived, and the impatient ones should turn with gratitude and admiration to the Royal Family of the nation, who had arranged to go down to their home in Norfolk and, like their subjects, from the humblest to the highest, spend a grateful and thankful Christmas, being the first after the Armistice. Every arrangement was made for the departure from London of Their Majesties, the King and Queen, Queen Alexandra, Princess Mary, and other members of the Royal Family, with the exception of the Prince of Wales and Prince Albert, the former of whom has gone to the Front to spend his Christmas with the Australian Forces, while Prince Albert has returned to the Royal Air Force, that he may enjoy Christmas and spend it with his fellow airmen. Almost at the last moment affairs of State have intervened, and Their Majesties the King and Queen, Queen Alexandra and the other members of the Royal Family have remained at the post of duty and will stay in London over Christmas. This must have entailed serious disappointment to all concerned, but Their Majesties will have the satisfaction of knowing that their people have long since gratefully recognised that our King and Queen have become the State's greatest public servants. Indeed, it is Their Majesties' service, of which the present is the latest and most remarkable exemplification, even more than their Royal station, that has become our King and Queen's claim to the devotion of the people of the Empire, whose attachment to them, and their pride in the noble example they have set, were so touchingly and universally exhibited when the Armistice was declared, and throughout the days which immediately followed that great event.

The war has made one material difference in the keeping of Christmas in many hospitals in the homeland. Last year we specially directed attention to the great opportunity which all hospital authorities had to provide that Christmas in the hospitals, and the days which immediately followed, should endure in the memory of every warrior, every civilian adult and child patient throughout the country. This invitation was cordially accepted by the authorities of very many hospitals from the oldest, largest, and most efficient, through almost every type of institution, including many of those which have been organised to meet the demands of the war. It was one of the most cheering and uplifting days in the whole year for very many members of the staff, and for our warrior patients, many of whom had never encountered such a Christmas Day before. They

☛ Previous articles appeared on July 28, August 11 and 18, September 1, December 29, 1917 and August 10, 1918, pp. 412-13.

**The Worst War of the World**—(*continued*).

entered wholeheartedly into the spirit of the occasion, and rendered material aid and additional charm to the decorations and contributed much of the humour introduced into the proceedings, so that all the patients had a blessed Christmas indeed. There were many instances where warrior patients declared that they had never expected to have such a day in a hospital or at all. It was just "Blighty" at its very best. The same feeling was universally experienced, we are glad to hear, by every hospital resident, including all grades, who gave of their best to make the end of the fighting year 1917 memorable, and fruitful, in good works.

This year the Armistice has come, and it has been said, with some force and truth, that the twenty-fifth December 1918 should be memorable as probably the greatest Christmas in the history of the world, having regard to the momentous events which have happened within the last few months, and the fact that the hour is approaching when negotiations will be entered into for the organisation of a lasting peace, won for humanity by the enforcement of Truth and Right, and the vindication of Civilisation and Justice for all, throughout the nations of the earth. Even those of us who have lost our nearest and dearest have given them out of full hearts because of the assurance that this war has been God's War. Out of it we may hope that humanity will find salvation for all, and that the world will enter upon a new era, freed from the old tyrannies, abuses, and militarism, in which every man, woman, and child, who is true-hearted and worthy of it, will enjoy freedom, protection, and the right to live. We have not lost our dear ones, for they have been glorified by their splendid heroism and voluntary sacrifice, that those for whom they fought and died might be freed from the horrors, cruelties, and slavery which their enemies planned to inflict upon them. We know in our hearts, and thank God for it, that all this noble sacrifice has not been in vain.

There has been a great awakening to the recognition of the spiritual nature of man and woman and child. We confess to the feeling that out of this will come for the people a new and a better world, wherein they can live their lives, help their fellows, and secure for their neighbours, and, in co-operation, for the free nations of humanity, opportunities, developments, and a wiser and better recognition of the glorious blessedness of being in tune with the infinite, and inspired by the spirit of resolution to do their best and give their best to make life on earth a season in which everybody will have full opportunity to co-operate with their neighbours to help their fellows, and to become better men and women, progressively, throughout their lives. It should mean the will to do their best, to shed self, and steadily settle down to develop to the fullest extent of the capacity of each in those directions and things which matter to men, women and children in this life, as a preparation for the life which is beyond. So Christmas 1918 may properly be regarded by us all as the Christmas of our lives, which will be full of inspiring memories, and quicken us for the new and better life which should come to humanity through the Peace which is ahead, resting on the triumph of civilisation over tyranny, militarism, outrage and wrong. It should mean the salvation of humanity and the willing enforcement throughout the world of Truth and Justice, clean lives and noble purposes so long as the world endures.

It is very grateful to remember and realise how fully the members of the Reigning House in this country have identified themselves with the uplifting, well-being, life and ambitions of the people. Events have proved that the people's cares and troubles, hardships and difficulties, sufferings and misfortunes, find the deepest and most sympathetic response in the hearts of our King and Queen, of Queen Alexandra, and the members of the Royal Family. We have preached the doctrine of personal service, and the joyousness which every true-hearted member of our nation experiences who devotes time and energies at Christmas to cheer and brighten the lot of the sufferers in our hospitals, to take them out of themselves and make Christmas Day for them not only a pleasing memory but an enduring gain. It is well known that their Majesties the King and Queen have, for a decade at least, deliberately given up certain days in each year to devote them to good works and personal service. Last Christmas Queen Alexandra and her devoted and much-honoured daughter, H.R.H. Princess Victoria, gave up a whole afternoon that they might spend it in the London Hospital, of which Her Majesty is president, with eight hundred children, many of whom had suffered from air-raids. These are happenings to remember and be grateful for.

All workers, and indeed every resident in these islands, our men who are absent on naval and military duty in connection with the war, and all the inhabitants of the Empire beyond the Seas, with our brothers the Americans, will gratefully appreciate the fact that the King and Royal Family have remained on duty throughout the whole of the Christmas Season this year at the call of the State. Every day, as the newspapers record, His Majesty the King's time is so fully occupied that the King is proved to be one of the hardest workers in the nation. There will be no Christmas holiday this year for the King and Queen. As the servants of the State they will be continuously engaged on Boxing Day and onwards, as representatives of the Empire and the Homeland, in extending the heartiest of welcomes to the President of the United States, and in the multifarious affairs which await their attention in the New Year. These must occupy the King until Peace is consummated, and it is possible to turn from the discharge of the business of the nation to the claims of private, family and home life, under the altered conditions created by the war. Henceforward the people should develop until all classes are united by a common agreement, that, a new world has opened to all. Then everybody can co-operate gladly in promoting everything best calculated to give the maximum of blessedness and satisfaction, with a lasting peace, to free men and women of good hearts and good will.

